# Symptoms of depression impact the course of lung function in adolescents and adults with cystic fibrosis

**DOI:** 10.1186/1471-2466-14-205

**Published:** 2014-12-16

**Authors:** Astrid Fidika, Marion Herle, Lutz Goldbeck

**Affiliations:** Department of Child and Adolescent Psychiatry/ Psychotherapy, University Ulm Medical Centre, Steinhoevelstr. 1, 89075 Ulm, Germany

**Keywords:** Cystic fibrosis, Depression, Lung function

## Abstract

**Background:**

Epidemiological studies report high rates of depression among patients with cystic fibrosis (CF). Assuming a causal relationship between depression and the progression of CF, our hypothesis is that elevated symptoms of depression would be a predictor of worse lung function after two years.

**Methods:**

In the context of the TIDES study, 473 German patients with CF (age 12–53 years, FEV_1_% predicted *M* = 66.2, range 13–137) completed the Hospital Anxiety and Depression Scale (HADS). Lung function (FEV_1_% predicted) was assessed at baseline and followed up two years later. Repeated measures analysis was performed involving the level of FEV_1_% and the level of depressive symptoms at baseline as independent factors and FEV_1_% at the 2-year follow-up as the dependent variable.

**Results:**

Interaction between lung function and depression at baseline significantly affected the change in lung function at the 2-years observation interval. The largest decline in FEV_1_% occurred in depressed patients with good lung function at baseline. In contrast, patients without any clinically relevant depressive symptoms and with poor lung function at baseline showed a slight increase two years later.

**Conclusion:**

The findings emphasise the need to screen patients with CF for symptoms of depression and to treat co-morbid depression.

## Background

Forced expiratory volume in one second (FEV_1_%) is the key outcome in cystic fibrosis (CF) for monitoring the course of the disease [1]. Average rates of annual decline in FEV_1_% are reported to be between one and three per cent [2, 3]. A range of biological risk factors such as infection with *Pseudomonas aeruginosa* or pancreatic insufficiency promote this decline [3–6]. Moreover, associations between pathophysiological processes, such as inflammation, and increased symptoms of depression were identified in the context of other medical conditions [7–9]. In addition, psychological factors might explain additional variance in the course of the disease, given their association with adherence to treatment.

Prevalence rates for depression range between 6.9% in Europe [10] and 8.6% in the US [11]. They therefore, point to a major public health issue worldwide. Recently, a German study found that nine per cent of adolescent and adult patients with CF show clinically relevant symptoms of depression, similar to the prevalence in the general population [12]. According to the international TIDES study involving nine countries [13], 10% of adolescents and 19% of adults with CF reported elevated symptoms of depression. It is important to address depression in the context of medical treatment for two reasons. First, depression is a recognised major mental health problem and is associated with poor quality of life [14]. Evidence-based treatments for depression are available [15, 16] and patients with CF should be given an opportunity to access these treatments. Depression is a known risk factor for poor adherence to treatment more particularly in the case of patients with chronic conditions [17]. Chronically ill patients who screened positive for symptoms of depression have a three-fold higher risk of not adhering to some degree to their medical treatment [18]. Symptoms of depression such as low mood, lack of energy or hopelessness may impair the patient’s willingness and ability to adhere to his medical treatment plan. Self-monitoring of CF-symptoms may be limited owing to depression and patients may find it difficult to identify situations where medical assessment and intervention would be appropriate. Poor adherence to the medical regime limits treatment effectiveness, promotes disease progression and may, ultimately, reduce survival [19].

Accordingly, previous research showed that the mental health state of patients with chronic lung diseases does indeed impact relevant medical outcomes. Patients with asthma or chronic obstructive pulmonary disease have better disease outcomes in the absence of co-morbid anxious or depressive disorders [20–22]. Likewise in patients with CF symptoms of depression are inversely associated with lung function [12, 22]. Hence, patients with severely impaired lung function reported the highest levels of symptoms of depression. In a recent study the decrease in lung function over a 12 year period was associated with a deterioration in health-related quality of life (HrQoL). This is known to be inversely correlated with depression [23]. Another study found that specific HRQoL domains, i.e. the pain domain in the SF-36 questionnaire and the physical functioning domain in the Cystic Fibrosis Quality of Life Questionnaire, contributed prospectively to the prediction of the survival of adults with CF [24].

Summarizing the evidence from epidemiological studies, it could be argued that progression of CF drives co-morbid depression. However, the association between depression and the course of the CF may be reciprocal but so far there have been no longitudinal studies that disentangled this relationship in patients with CF. To further investigate the association between depression and lung function in patients with CF, we prospectively examined the hypothesis that symptoms of depression would be a predictor lung function in a two-year follow-up assessment when controlling for baseline disease severity of the CF-related lung disease.

## Methods

### Participants and procedure

Self-reported symptoms of depression were assessed in the German arm of the international epidemiological study on depression and anxiety in patients with CF (TIDES) [12]. These data were used as baseline measures for the current longitudinal study. Medical data at baseline and two years later were retrieved from the patients’ medical charts as documented in the German CF registry.

All patients consented to participate in this TIDES study and to the use of their individual medical data from the German CF registry. The IRB at the University of Ulm approved the study.

612 of the 670 patients participating in the German arm of the TIDES study, were treated in German CF centres and their data were stored in the German registry. Complete baseline data, which included HADS depression score and lung function, were available for 575 of these patients. As follow-up data on lung function two years later were not available for all patients, the study sample for the current longitudinal analysis comprised 473 patients. No information was available about reasons for follow-up losses, such as death of a patient. No differences in FEV_1_% at baseline were observed between the patients with complete follow-up data and non-complete data (*t* = −1.61; *df* = 573; *p* = .108) or for symptoms of depression (*t* = −1.95; *df* = 573; *p* = .052).

### Measures

#### Demographic and FEV_1_% data

Demographic information (age, gender, professional status, and living situation) and FEV_1_% were retrieved from the annual reports of the CF clinics to the German CF registry. The FEV_1_% value closest to the patient’s birthday was used as the indicator of lung function.

#### Symptoms of depression

Symptoms of depression were measured using the depression subscale of the Hospital Anxiety and Depression Scale (HADS) [25], a brief and easy-to-administer screening tool for assessing symptoms of anxiety and depression in medical populations. Any physical symptoms of depression confounding with a chronic illness, such as fatigue or headaches, were excluded. For the prior seven-day period, patients rate the magnitude of each symptom of seven core symptoms of depression on a four-point scale ranging from 0 to 3. Raw scores are added and established cut-off scores distinguish between normal (0–7 points), borderline (8–10 points), and clinical symptom levels (≥11 points) [25, 26]. Many studies that use the HADS confirmed that it is valid for both clinical and community settings. Good psychometric properties have been demonstrated [26, 27] for the German version, too [28].

### Statistical analyses

First, all data were analysed descriptively. Frequencies of cases with elevated symptoms of depression using the established cut-off score (8 or higher) [25, 28] were computed.

To examine differences in change in lung function according to the status of depression and disease severity at baseline, a 2×2 repeated measures ANOVA was conducted with lung function at baseline (FEV_1_% ≤40% vs. >40%) and depression at baseline (HADS depression 0 to 7 vs. 8 or higher) as independent variables, and lung function (FEV_1_% predicted) at follow-up as a dependent variable. Cut-offs for classification of FEV_1_% as severely limited range from 30% to 50%. We chose the cut-off of ≤40%, because it indicates a significant airflow limitation and is commonly used in other studies. Effect sizes (*η*^*2*^) were computed (*η*^*2*^ > 0.01 indicating small, *η*^*2*^ > 0.10 medium, and *η*^*2*^ > 0.25 large effects) [29].

As regression to the mean (RTM) is a relevant cause of misinterpretation of results based on repeated measures, we investigated RTM quantification in accordance with Barnett *et al.* [30]. Because the group of patients with and the group without elevated symptoms of depression showed a significant difference in their mean FEV_1_% at baseline (*t* = 8.29; *p* < .001), we computed RTM effects separately for both groups and then checked these results against the means of the change scores (follow-up FEV_1_%- baseline FEV_1_%) of the four sub-groups.

All statistical analyses were performed with IBM Statistics SPSS 20.0 for Windows.

## Results

### Characteristics of the study sample

The age of the patients at baseline assessment ranged from 12 to 53 years (*M* = 23.2, *SD* = 8.6); 55.4% were male. Most of the patients were living with their family or their partner (79.3%) and were in education or employment (in school 27%, in job training 35.5%, employed 21.9%, unemployed 5.1%, retired 10.4%).

The patients’ lung function data at baseline are presented in Table 1. Altogether, 38 (8%) patients reported elevated levels of depression, with 21 (4.4%) of them in the borderline range at baseline. 89 (18.8%) patients had a severe lung disease at baseline (FEV_1_% ≤ 40). Patients with elevated symptoms of depression differed significantly in their FEV_1_% (depressed: *M* = 44.33, *SD* = 16.01 vs. not depressed: *M* = 68.06, *SD* = 24.39; *t* = 8.292; *p* < .001) from the patients with no elevated symptoms.

**Table 1:** **Lung function (FEV**_**1**_ **%) at baseline among the study sample and subgroups**

	Study sample (n = 473)	FEV _1_% ≤ 40%; depressed (n = 21)	FEV _1_% ≤ 40%; not depressed (n = 68)	FEV _1_% > 40%; depressed (n = 17)	FEV _1_% > 40%; not depressed (n = 367)
Range	13.7-137.3	23.1-39.0	13.7-39.7	40.4-76.5	40.1-137.3
M	66.2	32.2	31.4	59.6	74.8
SD	24.7	4.8	6.5	11.1	20.0
≤40%	89 (18.8%)				
>40%	384 (81.2%)				

### Depression and course of FEV_1_%

**Figure 1:**
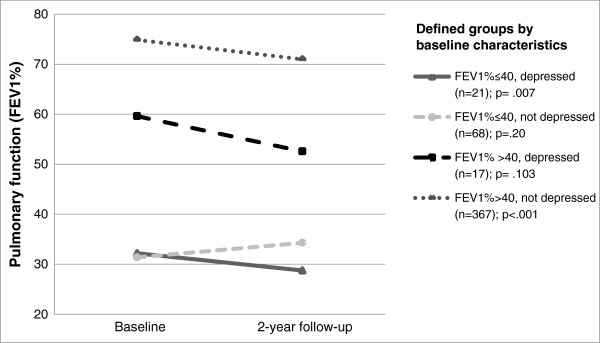
**Change of FEV**_**1**_ **% predicted over the period of two years, analyzed by level of lung function and state of depression at baseline (n = 473).**

As expected, a significant main effect of time (*F* = 5.96; *p* = .015; *η*^*2*^ = 0.013) indicated an average decrease of about 3% in the FEV_1_ predicted in our study sample during the two year observation period (Figure 1). A significant effect of group affiliation (*F* = 118.18; *p* < 0.001; *η*^*2*^ = 0.43) is incidental due to the fact that FEV_1_% predicted was one baseline criterion for classification.

A significant group x time interaction effect (*F* = 5.44; *p* = .001; *η*^*2*^ *=* 0.034) reveals that in the four groups lung function changed differently over time. The mean lung function decreased in three of the four groups. The largest group, i.e. patients with moderate to good lung function without symptoms of depression at baseline, declined from 74.9% (*SD* = 20.0) to 70.9% (*SD* = 22.1) during the two years under observation. Depressed patients with moderate or good lung function at baseline showed the greatest decline of all four groups from an average of 59.6% (*SD* = 11.1%) at baseline to 52.0% (*SD* = 20.7%) two years later. Patients with poor lung function and symptoms of depression at baseline decreased on average a further 3.5% from 32.2% (*SD* = 4.8) to 28.7% (*SD* = 4.9). Contrary to the expectations, severely ill patients without depression at baseline increased slightly from 31.2% (*SD* = 6.5) to 34.3% of the FEV_1_ predicted (*SD* = 17.09).

The estimated RTM effect was +/−1.06 for the groups of patients with symptoms of depression within the normal range (n = 435). It was +/− 2.66 for the group with elevated symptoms of depression (n = 38). The observed average changes within the groups were higher than this score for each of the four corresponding subgroups: FEV_1_% ≤ 40, depressed (*M* = −3.41; *SD* = 5.26), FEV_1_% ≤ 40, normal (*M* = 2.86; *SD* = 18.28), FEV_1_% > 40, depressed (*M* = −7.03; *SD* = 16.75), and FEV_1_% > 40, normal (*M* = −3.87; *SD* = 12.36).

## Discussion

This is the first study to examine the impact of depression on change in lung function, especially FEV_1_% predicted, in adolescents and adults with CF. The results show that clinically relevant symptoms of depression may increase the loss of lung function and that severely ill patients without depression are able to improve their lung function against the progressive trend of CF. Therefore, in a bio-psycho-social model depression should be considered as an important risk factor for disease progression in patients with CF. This backs the position that mental health is a relevant factor as already mentioned in the literature in conjunction with other chronic conditions that impair the respiratory system [20–22]. Given our prospective study design, assumptions may be made about causality. Moreover, the phenomenon of RTM in repeated measured data as a risk factor for misinterpreting results was taken into account. The estimated effects of RTM in the groups were lower than the observed changes in lung function. We can therefore, conclude that our findings are not solely based on RTM.

Our results are consistent with a theoretical model about the relationship between adherence, self-management behaviours and disease outcomes outlined by Modi *et al*. [31]. The interaction between depression and disease progression could be linked to the lack of hope, optimism and perceived self-efficacy in depressed patients, consequently inhibiting their fighting spirit against the disease and necessary adherence to treatment. Thus, depressed patients may fail to respond to standard medical treatment due to the nature of their mental health problems, and may experience a greater loss of lung function than necessary. This effect may then reinforce the negative thoughts related to their own prognosis, triggering a vicious psycho-somatic cycle. In contrast, patients who are not depressed and already have severely restricted lung function might be more able to make use of resources for their daily treatment routines. This, in turn, might result in stable or even slightly improved outcomes.

Despite the strengths of this study, such as the relatively large sample size and the prospective design allowing for causal conclusions, some limitations may have biased our results. Due to study participants who were lost for follow-up for unknown reasons, a selection bias may have occurred in our results. However, our subgroup comparisons in what is still a relatively large sample for a rare condition like CF constitute a sound empirical basis for our results. The collection of data was based on registry data which may lead to limitations in interpretation [32]. More particularly, information about lung function (FEV_1_% predicted) is not fully available over the two-year period in the registry because it is documented as a single-point measure in the observed year. This may cause higher individual variations than when using means or longitudinal data per year and does not allow for consideration of variability in patient lung function. Given the small number of patients in the groups, we were not able to control for confounders, such as CF-related diabetes or status of colonisation with germs. This, too, could perhaps had provided some substantial information to help explain the variance in the course of lung function within our sample. Due to the lack of feasibility in a routine clinical setting, symptoms of depression were assessed by self-reports only, and the persistence of these symptoms was not assessed. The use of HADS has been discussed controversially within the TIDES study [13]. The questionnaire was developed for use in medical settings and was deemed to be a valid and reliable screening measure when planning the TIDES study [26, 27]. However, more recent studies report several shortcomings, such as underestimation of symptoms of depression [33, 34]. Other valid and reliable screening questionnaires, such as PHQ-9 [33, 35], might be a better choice for future studies and for use as a screening tool, as recently recommended by the International Guidelines Committee on Mental Health for Patients with CF and Their Caregivers [36]. Additionally, the generalisation of our results to patients in other countries warrants future investigation.

## Conclusions

Our findings have several important clinical implications. Previous studies established that although there are substantial rates of co-morbid symptoms of depression in adolescent and adults with CF, not all patients receive treatment [12, 22]. The current findings indicate that it is important to identify depressed patients and to offer them mental healthcare. Systematic screening procedures to identify patients needing treatment for their depression should become an essential component in the care of patients with CF, as recently recommended by the International Guidelines Committee on Mental Healthcare for Patients with CF and Their Caregivers [36]. The Committee was sponsored by the European CF Society and the CF Foundation of the US and stressed the importance of targeting symptoms of depression and anxiety through appropriate intervention. Considering the importance of depression which is again emphasised by the results of the current study, treatment goals should include stabilisation of the patients' mood, behavioural activation, and the creation of a sense of self-efficacy.

Since no evidence-based interventions seeking to decrease the symptoms of depression specifically in patients with CF are available at the present time [22, 37], those psychotherapeutic and psychopharmacological treatments known to be effective for patients with a primary diagnosis of depression should also be applied to patients with CF. However, in psychopharmacological treatment, possible reciprocal effects between CF medication and medication for depression have to be considered. Research efforts are needed to identify effective strategies for the delivery of treatments for depression in patients with CF, and to evaluate their risks and benefits. Furthermore, strategies to help patients keep their lung function stable or even improve it might be an interesting topic for further studies in order to learn more about resources and successful coping strategies. Further research on the long-term effects of co-morbid depression and their impact on disease progression could be worth examining, for instance to establish whether they are of equal prognostic value for survival.
